# The Role of Semipurified Fractions Isolated from* Quercus infectoria* on Bone Metabolism by Using hFOB 1.19 Human Fetal Osteoblast Cell Model

**DOI:** 10.1155/2018/5319528

**Published:** 2018-05-13

**Authors:** Amira Raudhah Abdullah, Hermizi Hapidin, Hasmah Abdullah

**Affiliations:** ^1^Biomedicine Programme, School of Health Sciences, Universiti Sains Malaysia, 16150 Kubang Kerian, Kelantan, Malaysia; ^2^Environmental and Occupational Health Programme, School of Health Sciences, Universiti Sains Malaysia, 16150 Kubang Kerian, Kelantan, Malaysia

## Abstract

*Background. Quercus infectoria* (QI) is a plant used in traditional medicines in Asia. The plant was reported to contain various active phytochemical compounds that have potential to stimulate bone formation. However, the precise mechanism of the stimulation effect of QI on osteoblast has not been elucidated. The present study was carried out to isolate QI semipurified fractions from aqueous QI extract and to delineate the molecular mechanism of QI semipurified fraction that enhanced bone formation by using hFOB1.19 human fetal osteoblast cell model.* Methods.* Isolation of QI semipurified fractions was established by means of column chromatography and thin layer chromatography. Established QI semipurified fractions were identified using Liquid Chromatography-Mass Spectrometry (LC-MS). Cells were treated with derived QI semipurified fractions and investigated for mineralization deposition and protein expression level of BMP-2, Runx2, and OPN by ELISA followed gene expression analysis of BMP-2 and Runx2 by RT-PCR.* Results.* Column chromatography isolation and purification yield Fractions A, B, and C. LC-MS analysis reveals the presence of polyphenols in each fraction. Results show that QI semipurified fractions increased the activity and upregulated the gene expression of BMP-2 and Runx2 at day 1, day 3, and day 7. OPN activity increased in cells treated with QI semipurified fractions at day 1 and day 3. Meanwhile, at day 7, expression of OPN decreased in activity. Furthermore, the study showed that combination of Fractions A, B, and C with osteoporotic drug (pamidronate) further increased the activity and upregulated the gene expression of BMP-2 and Runx2.* Conclusions.* These findings demonstrated that polyphenols from semipurified fractions of QI enhanced bone formation through expression of the investigated bone-related marker that is its potential role when combined with readily available osteoporotic drug.

## 1. Background

Osteoporosis is a major health problem with significant health consequences that may increase with age. The development of osteoporosis is due to imbalance production between osteoblast and osteoclast which are characterized by reduced bone strength and low bone mass and resulting in an increased risk of fracture that is associated with increase in substantial morbidity, motility, and social cost [1]. Bisphosphonates are widely used drugs as standard treatment for prevention of fragility fractures [2]. Although it is proven that biophosphonates are effective by limiting bone loss, however, there is growing concern over long-term use of biophosphonates which are linked to severe suppression of bone turnover and pose side effects which include gastroesophageal irritation and osteonecrosis of the jaw (ONJ) [3–7]. Anabolic therapy could be a potential agent that can induce bone remodeling as well as bone formation.

Bone is an active tissue that undergoes constant remodeling in which old bone is degraded by osteoclasts and subsequently replaced with new bone formed by osteoblasts through bone remodeling process [8]. Therefore, osteoblasts are the key components of the bone multicellular unit and have a seminal role in bone remodeling [9]. Bone metabolism includes the steps of proliferation, differentiation, and mineralization which are controlled and regulated by various osteoblastogenic marker. Bone morphogenic protein-2 (BMP-2), a member of the transforming growth factor-*β* (TGF-*β*) super family, is the key marker in controlling bone remodeling pathway [10]. It promotes differentiation of mesenchymal cells into osteoblasts by controlling the expression and functions of Runt-related transcription factor 2 (Runx2) which also known as Cbfa1/Pebp2*α*A/AML3 [11] via the Smad signaling pathway [12, 13]. Osteopontin (OPN) on the other hand is a type of marker expressed during the early stage of active proliferation and expressed maximally during mineralization phase together with other several proteins [14]. Thus, quantitative or qualitative measurement of these important osteoblastogenic markers is the key into understanding bone metabolism.

*Quercus infectoria *is a small oak tree widely distributed in Greece, Asia Minor, Syria, and Iran [15]. The galls have been reported to be of great medicinal value and widely been used as traditional medicine mainly as astringent and as anti-inflammatory [16]. Pharmacological evaluation of galls has shown that they have astringent, antidiabetic, antibacterial [17, 18], antifungal [19] anti-inflammatory, antiosteoporotic [20], and wound healing properties [21–23]. Research has reported that the galls of QI contain mainly a mixture polyphenols: 50% to 70% that includes gallotannin, gallic acid, ellagic acid, starch, and glucose as principal constituents. The known constituents of QI galls also include methyl gallate syringic acid, *β*-sitosterol, amentoflavone, hexamethyl ether, isocryptomerin, methyl betulate, methyl oleanate, and hexagalloyl glucose [24–26].

Polyphenols have been reported in several researches to promote bone formation. Yamaguchi and Ma, 2001, reported in their research that polyphenols in food had a potent anabolic effect on bone calcification in the femoral-diaphyseal and metaphyseal tissues of rats* in vitro* [27]. Recent studies by Shen et al., 2008, prove that green tea polyphenols are promising agents for preventing bone loss in women [28]. Moreover, polyphenols derived from dried plum also have been reported to enhance osteoblast activity and function by upregulating Runx2, Osterix, and IGF-I expression [29]. Furthermore, a review by Hapidin et al., 2012, suggested that QI may have a potential anabolic effect on bone metabolism [30]. Based on a preliminary study conducted by Hapidin et al., 2015, the level of alkaline phosphates (ALP) of human osteoblast cell (hFOB1.19) increasing significantly after being treated with QI galls extract proves the ability of QI in modulating bone metabolism [20]. Thus, in this study, the semipurified fraction of QI was derived and investigated for its effect on regulation expression of BMP-2, Runx2, and Osteopontin activity as well as gene expression of BMP-2 and Runx2 during osteoblast proliferation, differentiation, and mineralization by comparing it to different control groups.

## 2. Materials and Method

### 2.1. Preparation of Aqueous QI Extract

QI galls were purchased from local market and grinded to obtain powdered form for preparation of aqueous extract. The galls were identified based on its morphology parameters such as external color, size, surface, texture, odour, taste, and thickness [31]. The aqueous extract produced by weighing 50 g of crude QI extract in 100 mL of sterile distilled water and refluxing in water bath at 50°C for 24 hours was then filtered and concentrated using rotary evaporator followed by freeze-drying to obtain powdered form.

### 2.2. Fractionation of Aqueous QI Extract

Flash column chromatography was performed with silica gel 60, 0.063–0.200 mm, 60 Å pore size, pH range of 6.5–7.5 (Merck Milipore) in glass columns sized 40 mm width and 500 mm length. Solvent mixture (Ethyl Acetate : Methanol : Acetonitrile : H2O); ratio (1 : 1 : 7 : 1) was prepared beforehand. Manually packed flash columns were packed by utilizing the slurry method. Sample was prepared by dissolving 7.5 g of aqueous QI extract in 95% ethanol. The solvent mix was continuously added as the final layer simultaneously with elution process until collections of fraction were accomplished. The fractions were collected in test tubes, graduated with the fraction volume, and visualized by spotting on TLC plates using established solvent system (Ethyl Acetate : Methanol : Acetonitrile : H2O); ratio (6 : 1.5 : 1.5 : 1). The plate was then visualized under UV lamp at wavelength 254 nm. The fraction with the same Rf values will be pooled together and each fraction be subjected to second column chromatography by using different solvent mix ratio (Ethyl Acetate : Methanol : Acetonitrile : H2O); ratio (5.5 : 1 : 2.5 : 1). The fraction with the same Rf value will again be pooled together and considered as final semipurified products [18, 32].

### 2.3. Identification of QI Semipurified Fractions by Liquid Chromatography-Mass Spectrometry (LC-MS)

A total of 2.5 mg/mL sample of each QI semipurified fractions was prepared in ultrapure water. The compound detection utilizes Agilent-1290 Infinity system coupled to Agilent-6520 Accurate-Mass Q-TOF mass-spectrometer with dual ESI. Compounds then searched with Metlin_AM_PCDL-N-130328.cdb database.

### 2.4. Cell Culture Condition

Human fetal osteoblast cell, hFOB1.19 (CRL-11372), was purchased from American Type Cell Culture (ATCC) (Manassas, USA). The cell was cultured in Dulbecco's Modified Eagle Medium, DMEM F-12 (Gibco, USA), and supplemented with fetal bovine serum (FBS) (Gibco, USA) and 1% penicillin-streptomycin (Gibco, USA) [21]. The cells were incubated in 5% CO^2^ incubator at 37°C. All cell culture work was maintained in a sterile condition by using aseptic technique and carried out in Biosafety Cabinets Class 2 (ESCO, Singapore).

### 2.5. Cell Viability Assay

Determination of cell viability and half maximal effective concentration (EC_50_) was carried out using MTT 3-(4,5-dimethylthiazol-2-yl)-2-,5-diphenyltetrazolium bromide assay. Cells were plated in 96-well plate at a density 5 × 10^3^ cells per well [33] and treated with different concentration of each treatment groups range from (0.01–99 *μ*g/mL). A total of 5 mg/mL MTT powder, (Nacalai Tesque, Japan) was prepared by diluting in phosphate buffer saline (PBS) (Gibco, USA). Freshly prepared MTT was added to each of the 96-well plate followed by incubation for 4 hours. Then, formazan salts were dissolved with 100 *μ*L dimethyl-sulphoxide (DMSO) (Nacalai Tesque, Japan) and the absorbance was determined at 570 nm in ELISA microplate reader. The graph of the percentage of viable cell versus log⁡10 concentration (*μ*g/mL) of each fraction was plotted using GraphPad Prism 6.0. The half maximal effective concentration (EC_50_) was determined from the plotted graph.

### 2.6. Treatments Groups

These experiments consist of 3 control groups, untreated control, osteoporotic drug control (pamidronate), and negative control (tamoxifen), and 2 treatment groups, QI semipurified fraction-treated groups (Fractions A, B, and C) and combined treatment of QI semipurified fraction with osteoporotic drug (Fractions A, B, and C with pamidronate). Pamidronate was chosen as osteoporotic drug control to represent osteoporotic drugs biophosphonate, whereas tamoxifen, a type of selective estrogen receptor modulators (SERM), was chosen as negative control due to its known negative effect on osteoblast cells in which it inhibited both human osteoclast and osteoblast formation and bone resorption [34, 35].

### 2.7. Cell Proliferation Analysis

Cells were seeded in 12-well plates with seeding density 0.1 × 10^6^ and left overnight for cell attachment. Attached cells were then treated with all treatments groups and counted at days 1, 3, and 7 of incubation period. The cell were trypsinized with 0.25% Trypsin EDTA (Gibco, USA) and incubated for 3 minutes for detachment of cell. Detachment process was stopped through addition of 500 *μ*L culture media. The suspension was transferred into centrifuge tube and centrifuged at 1500 rpm for 5 minutes. The pellet was then resuspended in 1 mL culture media and stained using Trypan Blue (Gibco, USA) which was then transferred into Countess Slide Chamber. The cell number was counted by using Countess Automated Cell Counter (Invitrogen, USA).

### 2.8. Mineralization Assay: von Kossa and Alizarin Red S Staining

Fixed cells were stained with Alizarin Red stain for 30 minutes to check for calcium deposition and von Kossa stain for 40 minutes to check for phosphate deposition. Stained cells were observed under inverted microscope (Carl Zeiss, USA) and photomicrographs captured using image analyser (Carl Zeiss, USA). Both staining techniques allow simultaneous evaluation of mineral distribution and inspection of fine structures by microscopy.

### 2.9. Determination of BMP-2, Runx2, and OPN Expression Level by ELISA

Sample collection was conducted at days 1, 3, and 7 of treatments for detection of Runx2 (Cusabio, USA), BMP-2 (Raybio, USA), and OPN (Cusabio, USA) expression levels using commercially available kits. The reagents provided were prepared as directed in the protocol sheets. Each set of standard and samples were plated into the 96-well microtiter plate precoated with specific antibody and incubated for 2 hours at 37°C. Prior to incubation, 1x prepared biotin antibody was added to each well and incubated for one hour. The solution was then discarded and the plate was washed with washing buffer followed by addition of 1x HRP-avidin solution. The plate was then incubated for one hour at 37°C followed by removal of solution and the washing steps. TMB Substrate reagents were added at the end of the protocol and the color change was observed when the stop solution was added. The plate was read at absorbance 450 nm. Triplicate values were obtained and calibrated against the plotted standard curve of each marker.

### 2.10. Real-Time PCR

RNA was isolated using commercially available kit (Total RNA Mini Kit, Geneaid). The integrity and purity of the total RNA were verified using Nanodrop Reader. Isolated RNA was reverse-transcribed with cDNA reverse transcriptase kit (Bioline, UK) according to the manufacturer's protocol. Each reverse-transcription reaction contains 2 *μ*g RNA. The cDNA was amplified with Sensifast Hi ROX SYBR Green Mix (Bioline, UK) using an ABI Step one plus (Applied Biosystem) RT-PCR machine to quantify the expression of osteoblastogenic genes BMP-2 and Runx2 [36]. Each of the primer efficiency was determined by plotting the standard curve of standard sample with five-point dilution and determining the *R*^2^ value of the standard curve. Values between 90% and 120% were considered efficient [37]. The cycling condition used for real-time PCR was as follows: 10 min at 50°C, followed by initial denaturation/enzyme activation for 2 min at 95°C and 40 cycles of denaturation/enzyme activation at 95°C for 5 s; lastly, annealing and elongation at 72°C for 10 s. Data analysis was carried out using the StepOne Software V2.2.2. The housekeeping gene, Glutaraldehyde Phosphate Dehydrogenase (GAPDH), was used as endogenous reference gene to normalize calculation by using the comparative Ct method.  Table 1 shows the primer sequence used in this study.

### 2.11. Statistical Analysis

Data were expressed as the mean (SEM) of *n* = 3 independent experiments. Statistical analysis was conducted by using SPSS (version 22.0 for windows). Data were tested for normality and homogeneity of variance. Statistical comparisons of the results were established using repeated-measures ANOVA analysis. Differences among groups were conducted by using multiple comparison post hoc tests. Values of *p* < 0.05 were considered to be statistically significant to the control and indicated by (*∗*).

## 3. Results

### 3.1. Identification of Compound by Liquid Chromatography-Mass Spectrometry (LC-MS)

**Figure 1:**
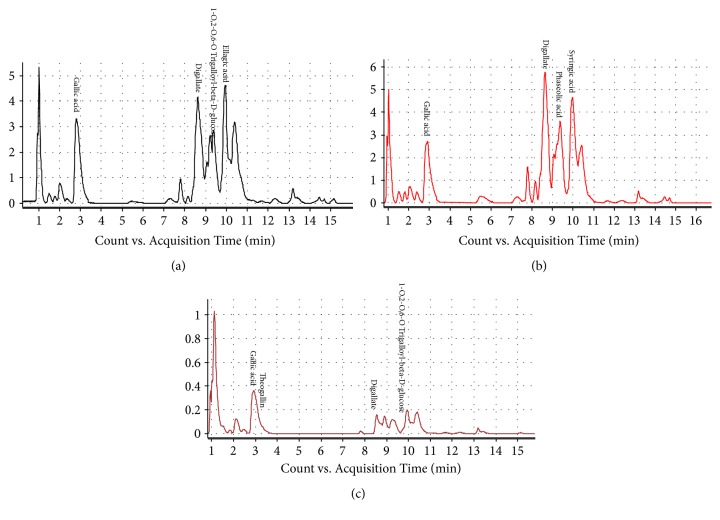
LC-MS chromatogram of QI semipurified fractions (a) Fraction A; (b) Fraction B; and (c) Fraction C. Conditions: Column Agilent Zorbax SB-C18, Narrow-Bonec (2.1 × 150 mm, 3.5 microns); temperature set at 25°C with (0.1% formic acid in acetonitrile) and (0.1% formic acid in water) as solvent system.

Fractions A, B, and C isolated from aqueous QI extract by means of column chromatography were each analysed by LC-MS to determine the composition of each compound presence in each fraction.  Figure 1 represents the chromatogram in each QI semipurified Fractions A, B, and C.

Semipurified fractions are generally crude extracts that have typically undergone further generic processing using a chemical filter to bias the screening profile which typically contains 1 to 5 compounds [38]. LC-MS findings (Table 2) indicate that each isolated fraction is made up from different composition of polyphenols each with different amount in (%). All fractions share similar polyphenolic composition gallic acid and digallate whereby Fraction C (65.88%) was detected with highest amount of gallic acid followed by Fraction B (62.1%) and Fraction A (40.95%). However, compound differentiating between Fractions A, B, and C are that Fraction A contains ellagic acid (6.22%); meanwhile, Fraction B contains syringic acid (24.73%) and Fraction C contains theogallin (8.17%), respectively.

### 3.2. Determination of Half Maximal Concentration (EC_50_) Dose Fixation by MTT Assay

**Figure 2:**
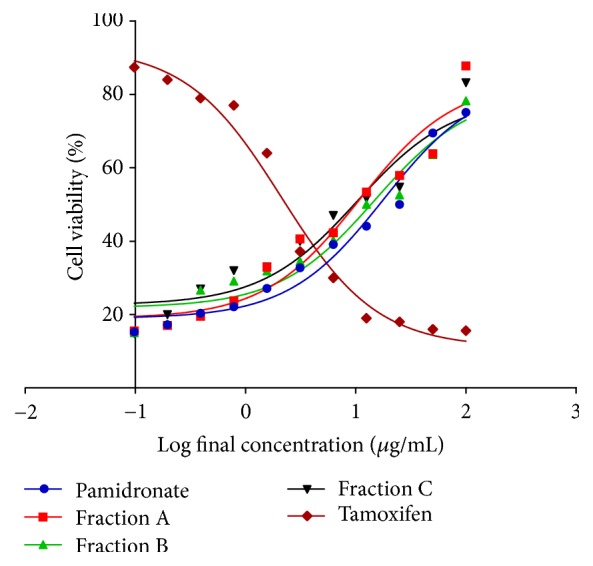
Graph of percentage of cell viability versus log final concentration of pamidronate, tamoxifen, and QI semipurified Fractions A, B, and C. Paired *t*-test was significant (*p* < 0.05). EC_50_ values were calculated by using dose-response stimulation curve in GraphPad Prism 6.0.

EC_50_ of control drugs and each QI semipurified Fractions A, B, and C were determined using MTT assay method. The established EC_50_ values are as follows: Fraction A, 10.85 *μ*g/mL; Fraction B, 12.00 *μ*g/mL; Fraction C, 11.60 *μ*g/mL; pamidronate, 15.27 *μ*g/mL; and tamoxifen, 1.568 *μ*g/mL, respectively. The percentage of cell viability (Figure 2) of pamidronate increased as concentration increased, whereby the percentages of cell viability of tamoxifen were the lowest at high drug concentration which exerts that tamoxifen inhibits cell proliferation at high concentration. Lower EC_50_ concentrations of Fractions A, B, and C are required to induce cell proliferation compare to osteoporotic drug control pamidronate. The cell viability of hFOB 1.19 cells treated with Fractions A, B, and C exerts increasing viability as the concentration increases and does not exert any inhibitory effects when compared with negative drug tamoxifen.

### 3.3. Effect of QI Semipurified Fractions on Cell Proliferation

**Figure 3:**
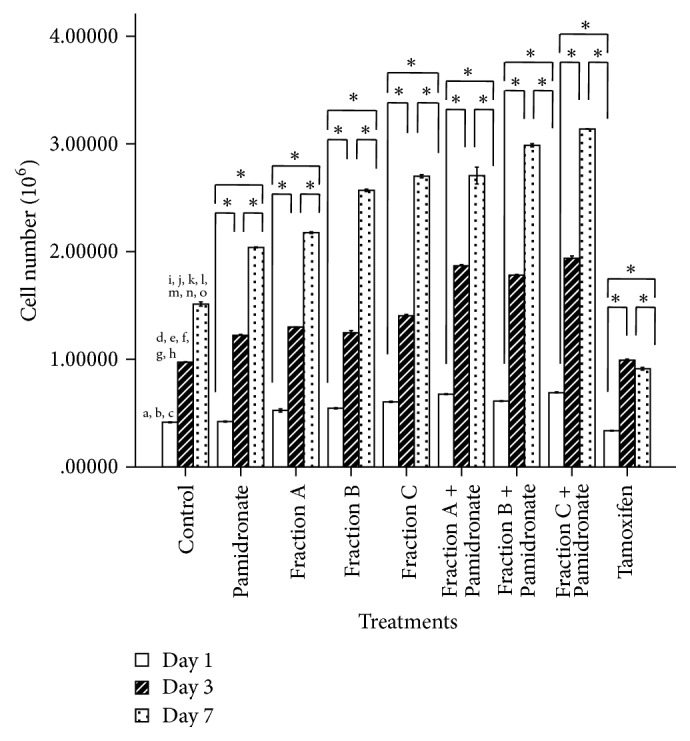
Cell number (×10^6^) after 1, 3, and 7 days of incubation. The values of the graph bar represent mean (SEM) of three independent experiments. *∗* indicates significant difference between three different days within the same groups (*p* < 0.05) whereas days that share similar letter show significant difference (*p* < 0.05), with different groups and similar days by repeated-measures ANOVA and Post Hoc test.

To examine the initial effects of the isolated QI semipurified Fractions A, B, and C on osteoblast model, cell proliferation was assessed between cell treated with QI semipurified Fractions A, B, and C treated group together with combined QI semipurified Fractions A, B, and C with osteoporotic drug pamidronate treated groups and compared among control, osteoporotic drug control (pamidronate), and negative control (tamoxifen) after 1, 3, and 7 days of incubation period. Results (Figure 3) show that proliferation of hFOB1.19 cells in all treatment groups except tamoxifen treated groups was significantly increased with time. After 24 hours of treatment (day 1) onwards, the proliferative activity of hFOB1.19 cells was significantly higher in QI semipurified Fractions A, B, and C treated groups compared to our control groups. Interestingly, the proliferation rate of hFOB 1.19 cells treated with combined treatment Fractions A, B, and C with pamidronate was more effective than QI semipurified fractions treated group without the presence of pamidronate. These results represent an early outlook on the potential combined treatment between QI semipurified fractions and osteoporotic drugs pamidronate which could lead to an efficient bone-forming agent.

### 3.4. hFOB.19 Cell Morphology and Mineralization Analysis after Alizarin Red and von Kossa Staining by Inverted Microscope

**Figure 4:**
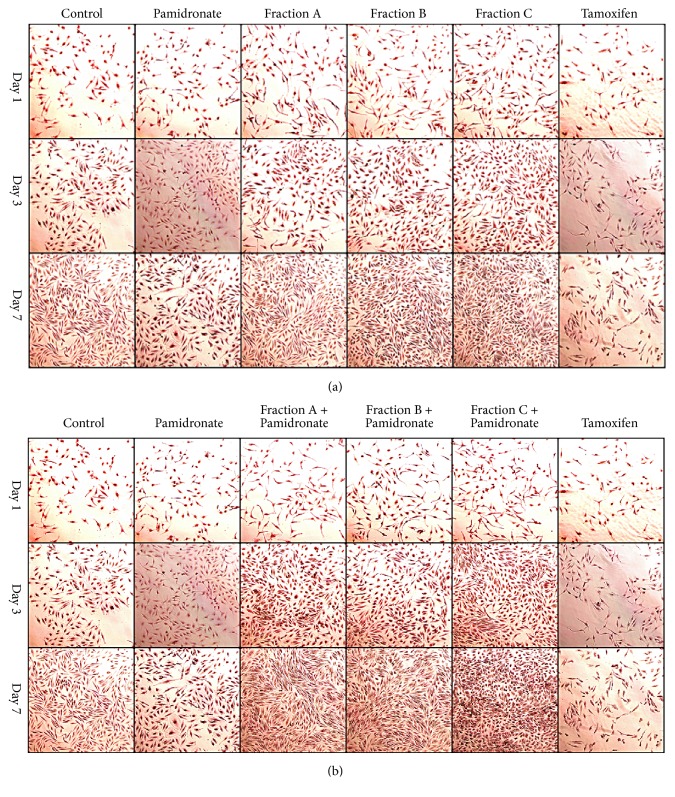
Alizarin red staining of hFOB 1.19 cell treated with (a) QI semipurified fraction-treated groups (Fraction A, Fraction B, and Fraction C); (b) combined treatment groups (Fractions A, B, and C with pamidronate) at days 1, 3, and 7 with comparison to control, osteoporotic control pamidronate, and negative control tamoxifen. Cell morphology was viewed using inverted microscope at 20x magnification and captured using image analyser. Calcium deposition appears as reddish black spot.

**Figure 5:**
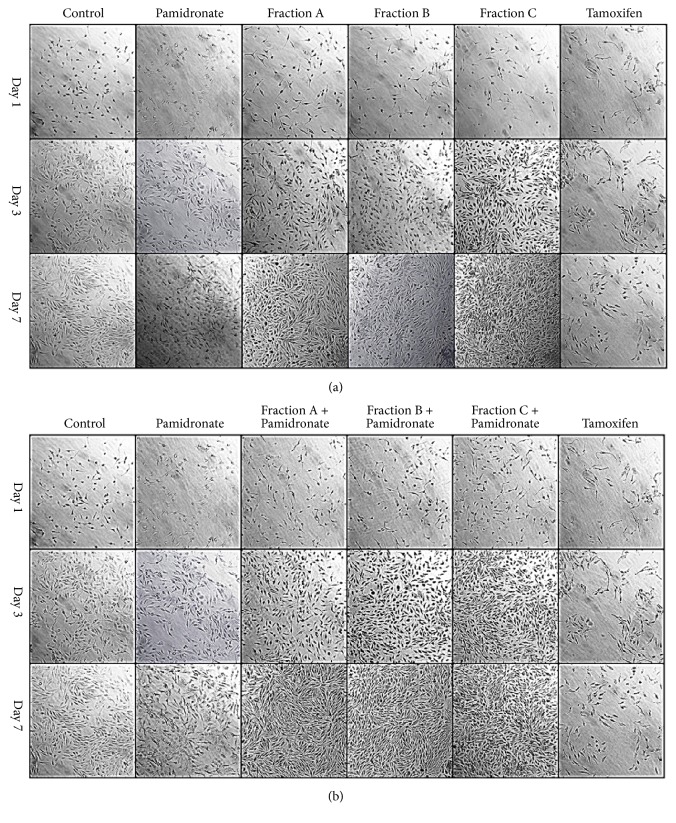
von Kossa staining of hFOB 1.19 cell treated with (a) QI semipurified fraction-treated groups (Fraction A, Fraction B, and Fraction C); (b) combined treatment groups (Fractions A, B, and C with pamidronate) at days 1, 3, and 7 with comparison to control, osteoporotic control pamidronate, and negative control tamoxifen. Cell morphology was viewed using inverted microscope at 20x magnification and captured using image analyser. Phosphate deposition appears as black spot.

In order to better understand the effects of QI semipurified Fractions A, B, and C and combined treatment of QI semipurified Fractions A, B, and C with pamidronate on hFOB 1.19 cells, the cell morphology and mineral deposition were assessed by Alizarin stain and von Kossa stain [39]. The Alizarin and von Kossa stain intensities were consistent with the results of proliferation analysis (Figure 3). As shown, cells treated with QI semipurified Fractions A, B, and C (Figure 4(a)), Alizarin stain (Figure 5(a)), and von Kossa stain after day 3 of incubation were observed to be uniformly elongated and slightly overlapping onto each other with slight calcium (reddish black spot) and phosphate (black spot) deposition. However, cells treated with combined treatment of QI semipurified Fractions A, B, and C with pamidronate (Figure 4(b)); Alizarin stain and (Figure 5(b)); von Kossa stain were observed to have a higher deposition of calcium and phosphate with higher confluency rate than cells treated with only QI semipurified Fractions A, B, and C. Meanwhile, cells treated with osteoporotic control pamidronate were observed to be more sparsely distributed with less calcium and phosphate deposition compared to the both QI-treated groups. In addition, cells treated with negative control tamoxifen show slight coloration to Alizarin Red and von Kossa stain and appear to be needle-like shape with sharp end, very sparsely distributed, and deteriorated.

At day 7 of treatment, cells treated with QI semipurified Fractions A, B, and C were observed to have higher intensity of reddish black spot indicative of calcium deposition (Figure 4(a)); Alizarin stain and black spot indicative of phosphate deposition (Figure 5(a)); von Kossa stain than day 3 and day 1. Moreover, QI semipurified Fractions A, B, and C treated cells also appear to be uniformly elongated, slightly rounded, and actively multiplying. On the other hand, cells treated with combined QI semipurified Fractions A, B, and C with pamidronate (Figure 4(b)); Alizarin stain; (Figure 5(b)); von Kossa stain were observed to be more actively multiplying compared to other treatment groups with highly abundant deposition of calcium and phosphate. However, cells treated with pamidronate at day 7 appear to be more elongated and less densely distributed and with signs of less calcium and phosphate deposition. As anticipated, cells treated with negative control tamoxifen were observed to be dead or deteriorated.

### 3.5. BMP-2, Runx2, and OPN Level of hFOB 1.19 Cells

**Figure 6:**
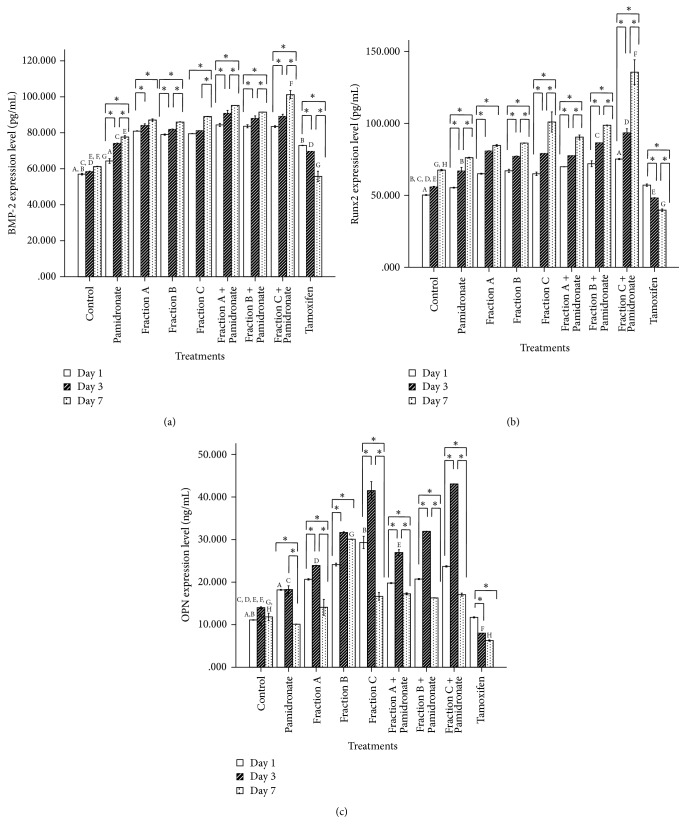
(a) BMP-2 expression level; (b) CBFA1/Runx2 expression level; and (c) OPN expression level expressed extracellular by hFOB 1.19 cells by control, pamidronate treated, QI semipurified Fractions A, B, and C treated, QI semipurified Fractions A, B, and C combined with pamidronate treated and tamoxifen treated after 1, 3, and 7 days of incubation. Values of the graph bar represent mean (SEM) of three independent experiments. *∗* indicates significant difference between three different days within the same groups (*p* < 0.05) whereas days that share similar letter show significant difference (*p* < 0.05), with different groups and similar days by repeated-measures ANOVA and Post Hoc test.

As mentioned earlier, in order to understand the mechanism of QI semipurified fractions acting on bone metabolism, we assessed the extracellular protein activities of selected osteoblastogenic marker that is involved in the process of osteoblast proliferation, differentiation, and mineralization, BMP-2, Runx2, and OPN by ELISA kit. Expression of BMP-2 and Runx2 (Figures 6(a) and 6(b)) in cells treated with QI semipurified Fractions A, B, and C and combined treatment of QI semipurified Fractions A, B, and C with pamidronate increased in a time-dependent manner prior to treatment at days 1, 3, and 7 which is contrary to expression of OPN (Figure 6(c)) whereby the expression of OPN increased at days 1 and 3 and decreased at day 7.

The finding also shows that hFOB 1.19 cells treated with combined treatment of Fractions A, B, and C with pamidronate expressed higher BMP-2, Runx2, and OPN than without pamidronate. In addition, the results show that hFOB 1.19 cells treated with Fraction C and combined treatment of Fraction C with pamidronate significantly expressed the highest level of BMP-2 and Runx2 at day 7 and OPN at day 3 of treatment.

### 3.6. QI Semipurified Fraction Upregulated Osteoblastogenic Gene

**Figure 7:**
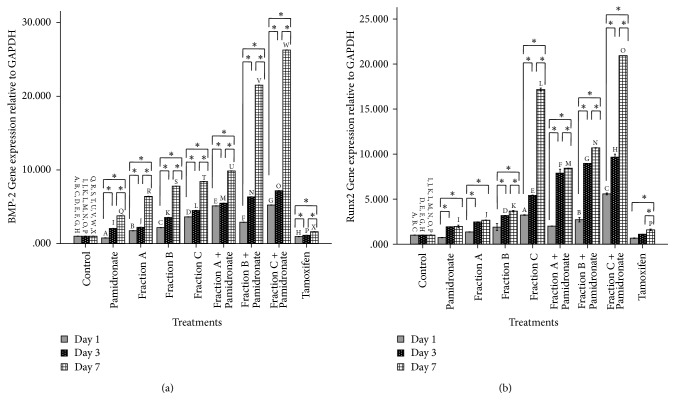
Relative gene expression analysis of osteoblastogenic markers at day 1, day 3, and day 7 in hFOB 1.19 cells expressed by control, pamidronate treated, QI semipurified Fractions A, B, and C treated, QI semipurified Fractions A, B, and C combined with pamidronate treated, and tamoxifen treated. cDNA of each samples was analysed by real-time PCR. The expression BMP-2 and Runx2, Figure 7(a). The gene expression of BMP-2 relative to GAPDH, Figure 7(b) The gene expression of Runx2 relative to GAPDH. All data are shown as mean (SEM) of three independent experiments. *∗* indicates significant difference between three different days within the same groups (*p* < 0.05) whereas days that share similar letter show significant difference (*p* < 0.05), with different groups and similar days by repeated-measures ANOVA and Post Hoc test.

To strengthen our findings, we also investigate the regulation gene expression of BMP-2 and Runx2 by real-time PCR. Results of relative gene expression are aligning with all the assays conducted earlier. Graph in Figures 7(a) and 7(b) shows that BMP-2 and Runx2 were upregulated in hFOB1.19 cells treated with QI semipurified Fractions A, B, and C in a time-dependent manner prior to treatment at days 1, 3, and 7. Meanwhile, similar to all assays performed, hFOB 1.19 cells treated with combined treatment of QI semipurified Fractions A, B, and C with pamidronate show higher upregulation of BMP-2 and Runx2 expression than cells treated without the presence of pamidronate. In addition, at day 7, both expressions of BMP-2 and Runx2 are upregulated the highest in hFOB 1.19 cells treated with Fraction C (up to 8.43-fold in BMP-2 and up to 17.1-fold in Runx2 relative to control) and combined treatment of Fraction C with pamidronate (up to 26.3-fold in BMP-2 and 20.9-fold in Runx2 relative to control). The present experiment suggested that Fraction C is a better choice of anabolic agent that helps to promote the process of bone metabolism in hFOB 1.19 cells through the upregulation of osteolastogenic markers BMP-2 and Runx2.

## 4. Discussion

This study evaluated the effects of established QI semipurified fractions (Fraction A, Fraction B, and Fraction C) on the biochemical analysis and gene expression of the important osteoblastogenic markers on the bone-forming cell, human osteoblast* in vitro *model (hFOB 1.19). Column chromatography, a widely known technique for establishment of desired phytochemical compound, was implemented in this study to obtain desired QI semipurified fractions using combination of different solvents in order to harvest the main polyphenolic content of QI galls [18, 40]. Each isolated fraction was then confirmed for the presence of polyphenolic compound by means of LC-MS. Different composition and amount of polyphenols presence in each fraction might explain the different efficacy effect of each fraction acting on hFOB 1.19 osteoblast cell model.

As mentioned above, polyphenols from different plants and sources have been reported in various studies and research to promote formation of bone by affecting bone metabolism [27–29]. Gallic acid and its derivatives are well established polyphenolic compounds that have been known to affect several pharmacological and biochemical pathways particularly in cancer therapy, immunomodulatory, and antioxidant research [41–43]. In this study, we are diverting our interest on gallic acid and its derivatives due to its tremendous medical benefits towards finding an alternative for osteoporotic therapy and bone-related disease. There are very few studies reporting the effect of these compounds on osteoblast [44]. To date, the effects of gallic acid derived from QI on hFOB1.9 osteoblast cell model have not been reported yet. Our findings reported gallic acid as the major component in each QI semipurified Fractions A, B, and C along with several mixture of other polyphenols. We believe that the presence of gallic acid in each fraction plays a major role in osteoblast metabolism, together with its interaction with different polyphenols in each QI semipurified fractions. Our results indicated that the gallic acid could be the vital compound that causes the enhancement of bone formation and mineralization as discussed in detail below.

As previously stated, the growing concern over osteoporotic therapy is due to most of available osteoporotic drugs acting as resorptive inhibitors which shows a trivial capability to enhance production of new bone [33]. Hence, there is a crucial need to explore other potential anabolic agent that can act as a suitable agent to rectify the imbalance in the process of bone remodeling. Since natural product is well known substitute for readily available drugs, the richness of phytochemical compounds isolated from QI may be an effective agent for osteoporotic therapy. Our study has interestingly shown that EC_50_ of QI semipurified fractions (Fraction A = 10.85 *μ*g/ml, Fraction B = 12.00 *μ*g/ml, and Fraction C = 11.60 *μ*g/ml) needed to induce cell proliferation which is lower than required by our osteoporotic drug control, pamidronate (15.27 *μ*g/mL). Meanwhile, the results of EC_50_ treatments with negative control drug were as follows: tamoxifen shows an inhibitory effect on hFOB1.19 cell at high drug concentration contrary to other treatments groups. This viability study may indicate that the QI semipurified Fractions A, B, and C derived from QI galls can facilitate osteoblast proliferation, differentiation, and adhesion whereas hFOB 1.19 cells treated with pamidronate were not proliferating and differentiating as effective as hFOB 1.19 cells treated with QI semipurified fractions.

Throughout the experiments, hFOB1.19 cells were treated with all three QI semipurified Fractions A, B, and C as well as combination of osteoporotic drug pamidronate with Fractions A, B, and C. Interestingly, after performing proliferative assay on all treatment groups and compared with the control groups, we found that the proliferative activity of hFOB 1.19 cells treated with combination of Fractions A, B, and C with pamidronate is better than cells treated with individual treatment of Fractions A, B, and C in comparison to pamidronate. We also found that individual treatment of hFOB1.19 cells with Fraction C shows a better proliferative activity than Fraction A and B. Likewise, results from proliferative study also indicate that combined treatment of Fraction C and pamidronate exerts highest proliferative activity compared to other treatment group. This shows suggestive evidence that active compound presence in Fraction C helps to enhance osteoblast proliferative activity and provide initial insight into the ability of combination treatment between QI semipurified fraction and pamidronate in promoting bone metabolism.

In order to support biochemical evidence, Alizarin and von Kossa stain were executed to study the morphological changes and mineral deposition of calcium and phosphate of treated cells. Results of the morphological study and mineral deposition study match our previous biochemical data. The key morphological characteristic of osteoblasts is that they are flat, spindle-shaped, and elongated with only slight areas of spreading at the end [45]. Following treatments with different treatment groups, hFOB 1.19 cell experiences morphological changes. The hFOB 1.19 cells that were treated with QI semipurified Fractions A, B, and C were observed to be well attached and have a uniformly elongated shape, parallel orientation and slightly rounded [46, 47] and located very close to each other due to rapid cell proliferation. Meanwhile, deposition of calcium (reddish black spot) and phosphate (black spot) is observed to be more abundant than all control groups. On the other hand, the hFOB 1.19 cells treated with combined QI semipurified Fractions A, B, and C with pamidronate were observed to be also well attached and have elongated shape and appear to be overlap on each other due to more rapid cell proliferation. Interestingly, it is found that the rate of depositions of calcium and phosphate on hFOB 1.19 cells treated with combination of Fractions A, B, and C with pamidronate are better than cells treated with only Fractions A, B, and C alone in comparison to the control groups.

Meanwhile, hFOB1.19 cells treated with osteoporotic drug (pamidronate) appear to be sparsely distributed and less dense and observed to have less calcium and phosphate depositions. In addition, some parts of the cells were observed to be deteriorated as long-term use of pamidronate may reduce bone growth [4]. Conversely, cells treated with negative control tamoxifen are observed to have needle-like shape with sharp end with the cell being very sparsely distributed, whereby the calcium and phosphate deposition only leaves a small trace which indicates very little to no mineralization deposition. The cells treated with tamoxifen were either dead or deteriorated as time increases. Consequently, this study provides a distinct data suggesting that semipurified fractions derived from QI galls have the ability to improve cells attachment and adhesion. These characteristics make QI semipurified fractions be a promising anabolic agent for regeneration medicine.

BMP-2 promotes differentiation of mesenchymal cells into osteoblasts by controlling the expression and functions of Runx2 via the Smad signaling pathway [12]. OPN is expressed during the early stage of active proliferation and expressed maximally during mineralization phase together with other several proteins [48, 49]. The expression of BMP-2, Runx2, and OPN in hFOB1.19 cells treated with all treatment groups will provide us with an insight into the effect of QI semipurified fractions on bone metabolism. Expression of BMP-2 and Runx2 in cells treated with Fractions A, B, and C as well as combined treatment of Fractions A, B, and C with pamidronate increased in a time-dependent manner prior to treatment at days 1, 3, and 7 which is contrary to OPN, whereby the expression of OPN increased at days 1 and 3 and decreased at day 7. We believe that decrease in OPN expression can be explained by Huang et al., 2004, in their research whereby it is reported that BMP-2 marker does not only stimulate expression of other bone-related marker but also reduces OPN signal [37]. However, due to sensitivity of OPN expression and different type of osteoblast cell line used, the time reported in each finding may be different. The finding also shows that hFOB 1.19 cells treated with combined treatment of Fractions A, B, and C with pamidronate expressing higher bone markers (BMP-2, Runx2, and OPN) than without pamidronate. In addition, congruent to our primary biochemical findings, hFOB 1.19 cells treated with Fraction C and combined treatment of Fraction C with pamidronate significantly expressed the highest level of BMP-2 and Runx2 at day 7 and OPN at day 3 of treatment.

In order to support our finding, we perform real-time PCR analysis to quantify the gene expression of BMP-2 and Runx2 expressed by hFOB 1.19 cells treated with all treatment groups. Our results demonstrate that BMP-2 and Runx2 gene expressions are upregulated in the QI semipurified fractions and QI semipurified fractions combined with pamidronate treated group similar to our results on extracellular protein. BMP-2 is essential in the regulation of bone proliferation, maintenance, and repair whereby it has the ability to induce osteoblast proliferation and differentiation in various type cells [50]. As mentioned, BMP-2 regulation is mediated by a receptor known as threonine kinase receptor and smad1 transcription factor in which these transcription factors are phosphorylated and form a complex with a common mediator known as smad4 and subsequently these complexes will be translocated into the nucleus in order to activate the regulation of Runx2 [51]. Runx2 has the ability to bind to the osteoblast specific cis-acting element (OSE) 2 in the promoter region of osteogenic gene [11]. Thereby, importance of Runx2 in osteoblast differentiation is very well established [10, 33].

Polyphenolic content isolated from QI galls presence in QI semipurified Fractions A, B, and C clearly demonstrated that they have the ability to promote and modulate the process of bone formation through regulation of important osteoblastogenic marker BMP-2, Runx2, and OPN in hFOB 1.19 cells. Fraction C is proven to be the most effective agent in promoting bone formation when compared to Fraction A and Fraction B. We believe that high percentage of gallic acid content in Fraction C is the contributing factor in making it the most efficient agent in this study. Surprisingly, our study has also demonstrated that the use of QI semipurified fractions combined with osteoporotic drug pamidronate is a more efficient agent to help induce bone formation, at the same time increasing the efficiency of pamidronate acting on osteoblast cell. Further and detailed studies are required to elucidate the mechanism by which QI semipurified fraction can help to reduce bone loss.

## Figures and Tables

**Table 1:** Primer sequence used in quantitative RT-PCR analysis.

Gene	Sense (5′-3′)	Antisense (5′-3′)
BMP-2	CGCTGTCTTCTAGCGTTGCT	GGGGTGGGTCTCTGTTTCAG
Runx2	GTGGACGAGGCAAGAGTTT	TACTGGGATGAGGAATGCG
GAPDH	GAAGGTGAAGGTCGGAGTC	GAAGATGGTGATGGGATTTC

**Table 2:** List of compounds and amount of compounds present in each of the QI semipurified Fractions A, B, and C based on LC-MS analysis.

Types of Fraction	Compound	Formula	RT	Mass	Percentage (%) volume
Fraction A	Gallic acid	C7H6O5	2.841	170.0215	40.95
	Digallate	C14H10O9	8.917	322.0325	28.78
	Ellagic acid	C14H6O8	10.177	302.062	6.22
	1-O,2-O,6-O-Trigalloyl-beta-D-glucose	C20H20O14	9.359	636.0963	24.05

Fraction B	Gallic acid	C7H6O5	2.877	170.0015	62.1
	Digallate	C14H10O9	8.916	322.0325	3.56
	Syringic acid	C9H10O5	10.055	198.0528	24.73
	Phaseolic acid	C13H12O8	9.476	296.0532	2.17

Fraction C	Gallic acid	C7H6O5	2.927	170.0215	65.88
	Digallate	C14H10O9	8.586	322.0325	22.71
	Theogallin	C14H16O10	3.135	344.0743	8.17
	1-O, 2-O, 6-O-Trigalloyl-beta-D-glucose	C20H20O14	9.366	636.0963	3.24
